# Hoxb4 upregulation by Xuan Bi Tong Yu Fang confers cardioprotection via repression of the Wnt/β-catenin pathway in myocardial ischemia-reperfusion injury

**DOI:** 10.3389/fimmu.2026.1767595

**Published:** 2026-05-07

**Authors:** Peng-fei Li, Han-ying Xu, Tian-ying Liu, Xiao-hui Li, Hong-yu Li, Guang-yu Cheng, Ai-dong Liu, Shuang-di Li

**Affiliations:** 1 Department of Nephrology, Affiliated Hospital of Changchun University of Chinese Medicine, Changchun, China; 2 College of Traditional Chinese Medicine, Changchun University of Chinese Medicine, Changchun, China; 3 Department of Cardiology, the First Affiliated Hospital of Henan University of Chinese Medicine, Zhengzhou, China; 4 Department of Cardiology, the Third Affiliated Hospital of Changchun University of Chinese Medicine, Changchun, China; 5 Research Center of Traditional Chinese Medicine, Affiliated Hospital of Changchun University of Chinese Medicine, Changchun, China; 6 Department of Cardiology, Affiliated Hospital of Changchun University of Chinese Medicine, Changchun, China

**Keywords:** HoxB4, myocardial ischemia reperfusion injury, traditional chinese medicine, Wnt/β-catenin signaling pathway, XBTYF

## Abstract

**Background:**

Myocardial ischemia-reperfusion injury (MIRI) remains a significant challenge in treating cardiovascular diseases, contributing to substantial myocardial damage and a poor prognosis. Traditional Chinese medicine (TCM) offers promising alternatives, with Xuan Bi Tong Yu Fang (XBTYF) being a classical formula known for its protective effects. However, the therapeutic mechanisms of XBTYF in MIRI have not been fully explored.

**Materials and methods:**

MIRI was induced in 36 Wistar rats, which were randomly assigned to sham, model, XBTYF (high, medium, low dose), and positive control (Qishen Yiqi Droplet) groups (n = 6). Serum levels of CK, AST, and cTnI were measured, and myocardial injury was examined by TTC, HE, and Masson staining. Bulk RNA-seq was performed to analyze gene expression profiles. The chemical composition of XBTYF was characterized by UPLC-MS/MS, followed by molecular docking to assess interactions between key compounds and Hoxb4. *In vitro*, H9C2 cardiomyocytes were subjected to oxygen-glucose deprivation/reoxygenation (OGD/R) and treated with XBTYF. The role of Hoxb4 in regulating the Wnt/β-catenin pathway was examined through gain- and loss-of-function approaches, together with the Wnt inhibitor IWR-1. Cell viability, proliferation, apoptosis, and the expression of apoptosis-related proteins and Wnt/β-catenin pathway components were assessed.

**Results:**

XBTYF treatment significantly reduced myocardial enzyme levels and myocardial infarction area in the MIRI rats. Histological analysis showed improved myocardial tissue structure and reduced fibrosis. Bulk RNA-seq analysis identified Hoxb4 as a key gene restored by XBTYF and revealed a significant suppressive effect of XBTYF on the Wnt/β-catenin signaling pathway. Chemical analysis identified multiple bioactive compounds in XBTYF, and molecular docking further revealed that ginsenoside Rg3, a primary component, exhibited a strong binding affinity with the Hoxb4 protein. *In vitro* studies confirmed that XBTYF upregulated Hoxb4, promoting cell proliferation and inhibiting apoptosis under OGD/R condition. Western blot analysis validated that XBTYF inhibited Wnt/β-catenin signaling, which was associated with reduced apoptosis and improved myocardial protection.

**Conclusion:**

XBTYF alleviates MIRI by upregulating Hoxb4 and subsequent inhibition of the Wnt/β-catenin signaling pathway, leading to reduced cardiomyocyte apoptosis and providing cardio protection. Its effects may be linked to direct interactions between bioactive constituents, such as ginsenoside Rg3, and Hoxb4. Collectively, these findings provide mechanistic and experimental evidence supporting XBTYF as a potential adjunctive therapeutic strategy for ischemia-reperfusion-related myocardial injury and establish a molecular basis for its further translational investigation and clinical application.

## 1 Introduction

Cardiovascular disease (CVD) remains a leading cause of mortality and disability worldwide, posing a substantial public health burden due to its high prevalence and fatality. According to the World Health Organization, CVD accounts for more than 17 million deaths annually, representing approximately 37% of all global deaths (1). This broad category encompasses coronary artery disease, heart failure, arrhythmias, and cardiac arrest. The heterogeneity and complexity of its underlying mechanisms not only complicate clinical management but also impose considerable economic strain on healthcare systems (2, 3).

Myocardial ischemia-reperfusion injury (MIRI) is a common and unresolved complication in the treatment of CVD, particularly during myocardial infarction and subsequent reperfusion. MIRI occurs when blood flow restoration causes injury to cardiac myocytes during reperfusion (4). Although reperfusion restores heart blood supply and reduces infarction, MIRI introduces a new challenge. Current therapeutic strategies, including pharmacological agents and mechanical interventions, remain suboptimal. Drugs such as antioxidants, calcium channel blockers, anti-inflammatory agents, and apoptosis inhibitors typically target single pathological processes and are often associated with limited efficacy or adverse effects. Mechanical interventions, including controlling blood flow rate and capacity and specific reperfusion strategies, are complex with unsatisfactory results (5). These limitations highlight the need for therapeutic strategies capable of targeting multiple pathways simultaneously.

Traditional Chinese medicine (TCM) has developed a unique system of treatments over thousands of years. In CVD, TCM offers a distinctive advantage and serves as an important supplementary method (6, 7). Recent research shows that traditional medicine offers considerable protection in MIRI surgery. Active ingredients in monomers or herbal compound regulate mechanisms such as antioxidant, anti-inflammatory, anti-apoptosis to protect the myocardium. For example, Tanshinone IIA from Salvia miltiorrhiza suppresses ferroptosis and apoptosis via voltage-dependent anion channel 1 (VDAC1) (8). Shenlian extract reduces miR-155 expression, thereby inhibiting M1 macrophage polarization and inflammatory responses (9). Astragaloside IV in Astragalus membranaceus regulates calcium ion balance and suppresses cell apoptosis (10), while notoginsenosides from Panax notoginseng improve cardiomyocyte function through multiple mechanisms (11–13).

Xuan Bi Tong Yu Fang (XBTYF) is a classical TCM formula used in the treatment of CVD. It consists of Ren Shen (Ginseng Radix et Rhizoma), San Qi (Notoginseng Radix), Yan Hu Suo (Corydalis Rhizoma), Chuan Xiong (Chuanxiong Rhizoma), Yu Jin (Curcumae Radix), and Bing Pian (Borneolum Syntheticum). The pharmacological activities of these individual components in the context of MIRI have been partially characterized. Ren Shen contains ginsenosides (e.g., Rg3, Rb3), which have been shown to attenuate post-ischemic apoptosis and inflammation, preserving ventricular function after MIRI (14, 15). Notoginsenosides from San Qi mitigate oxidative stress and calcium overload (16). Yan Hu Suo contains multiple bioactive alkaloids. Notably, dehydrocorydaline (Deh), an alkaloid isolated from Corydalis, has been demonstrated to protect against sepsis-mediated myocardial injury by inhibiting the TRAF6/NF-KB pathway, revealing potent anti-inflammatory and anti-apoptotic activities relevant to ischemic injury (17). Chuan Xiong, Yu Jin, and Bing Pian further support the formula by promoting blood circulation and enhancing bioavailability. Our previous studies have indicated that XBTYF alleviates mitochondrial damage, reduces cardiomyocyte apoptosis, and promotes angiogenesis, thereby conferring considerable myocardial protection in MIRI rat models (18). Nevertheless, its overall therapeutic effects and underlying molecular mechanisms remain incompletely defined. Therefore, the present study aimed to further elucidate the role and mechanism of XBTYF in MIRI, with the goal of providing experimental evidence to support its clinical application.

## 2 Materials and methods

### 2.1 Preparing XBTYF aqueous extract

The six component herbs of XBTYF were purchased from the First Affiliated Hospital of Changchun University of Chinese Medicine. This formula represents a classic prescription in TCM for the treatment of cardiovascular diseases, comprising six medicinal herbs, all of which are documented in the Chinese Pharmacopoeia (2025 edition). The aqueous extract was prepared at the experimental center by soaking the herbs for 30 min. The herbs were decocted three times using six volume of water, each for 30 min on low heat. The three aqueous extracts were mixed, filtered through gauze, and stored in a refrigerator at 4 °C.

### 2.2 Reagents

Hematoxylin (G1140), eosin (E8090), neutral resin solution (G8590), and PBS (P1010) were purchased from Wuhan Lingsi (Wuhan, China). The BCA Protein Assay Kit (G3422) was purchased from GBCBIO (Guangzhou, China). Trise-Base (1115GR500) was purchased from Biofroxx (Einhausen, German). Trypsin (BL501A), penicillin (BL505A), streptomycin (BL505A), and CCK-8(BS350B) were purchased from Biosharp (Hefei, Anhui). Fetal bovine serum (FBS, FB15015) was purchased CLARK (Virginia, USA). Total RNA Extractor (CW0597S) was purchased from Cwbio (Jiang Su, China). Reverse transcription and SYBR Green PCR kits (AQ131) were purchased from Full Gold (Beijing, China). The 5-ethynyl-2’-deoxyuridine (EdU) cell proliferation assay kit (C0071S) and phenylmethanesulfonyl fluoride (ST506) were purchased from Beyotime (Shanghai, China). Enhanced chemiluminescence solution (PE0010) and the Calcein-AM/PI Double Stain Kit (CA1630) were purchased from Solarbio (Beijing, China). IWR-1 (HY-12238) was purchased from MCE (New Jersey, USA).

### 2.3 Analysis of the chemical constituents of XBTYF

Three separate freeze-dried batches of XBTYF were subjected to analysis. The solvents and additives used included LC-MS-grade methanol, acetonitrile, ammonium acetate, and aqueous ammonia. Metabolite analysis was conducted on a Vanquish UHPLC system coupled with an Orbitrap Exploris 120 mass spectrometer (Thermo). Separation was achieved using a Hypersil GOLD C18 column (100 × 2.1 mm, 1.9 µm) held at 40 °C, operating at a flow rate of 200 μL/min. For positive ion detection, the mobile phase consisted of water containing 0.1% phase A formic acid and phase B methanol. The elution program (A/B, % v/v) was 0-1.5 min 98/2, 1.5–3 min linear to 15/85, 3–10 min linear to 0/100, 10-10.1 min return to 98/2, hold to 12 min. The mass spectrometer operated in data-dependent MS² over m/z 100–1500 with spray voltage 3.5 kV, sheath/aux gas 35/10 arbitrary units, capillary 320 °C, auxiliary heater 350 °C and S-lens RF 60. Rawdata were processed in Compound Discoverer 3.3 using 5 ppm mass tolerance for peak picking, retention-time alignment, blank subtraction and first-injection QC normalization; features with coefficient of variation > 30% were removed. Formula assignments used mzCloud, mzVault and an in-house MassList. After total-ion-current normalization, data were log-transformed and Pareto-scaled with metaX (R); principal-component and partial-least-squares discriminant analyses were performed, and metabolites with VIP > 1, Student’s t-test p < 0.05 and |fold-change| ≥ 2 were considered significant. Volcano plots, hierarchical-clustering heatmaps, Pearson-correlation networks and KEGG pathway enrichment were generated in R.

### 2.4 Animal group and model establishment

SPF-grade Wistar rats aged 6 to 7 weeks were obtained from the Hubei Province Experimental Animal Research Center (No. SCXK 2020-0018). The rats were reared at 22-26 °C and relative humidity 50-60%. After 7 d of feeding, 36 rats were divided into sham, model, XBTYF (high, medium, low dose), positive control (Qishen Yiqi Droplet), a total of 6 groups.

Rats were anesthetized with inhaled isoflurane and secured in the supine position on a dissection board. After shaving and disinfecting the neck, a midline cervical incision was made. The subcutaneous tissue and muscles were bluntly dissected to expose the trachea. An opening of approximately one-third of a tracheal ring was made on the anterior tracheal wall, avoiding blood vessels, and a tracheal cannula was inserted and connected to a ventilator for mechanical ventilation. A transverse incision was then made at the point of maximal cardiac impulse on the left chest. The muscles and intercostal tissues were carefully separated layer by layer, with gentle elevation of the ribs to avoid lung injury. The intercostal space was retracted using an eyelid retractor, and the pericardium was opened. The left anterior descending (LAD) coronary artery was identified along the left atrial appendage and ligated approximately 2 mm below its lower margin. Successful ligation was confirmed by blanching of the apical myocardium; if no color change was observed, the ligature was removed and repeated. After ligation, the thoracic cavity was cleared with sterile cotton swabs, and 2% lidocaine hydrochloride was applied to the cardiac surface to prevent arrhythmia. The intercostal space, muscles, and subcutaneous tissues were then sutured layer by layer. A chest drainage tube was placed, and residual air was aspirated before its removal. Finally, the cervical incision was closed. In the sham group, thoracotomy was performed without LAD ligation, while all other procedures were identical to those in the model group. Rats with successful modeling were randomly assigned to the model group, positive control group (Qishen Yiqi Droplet), and XBTYF low-, medium-, and high-dose groups. The XBTYF groups received 14 d of oral gavage at doses of 3.2, 1.6, and 0.8 g/kg/d (18). The positive control group (Qishen Yiqi Droplet) received 0.135 g/kg/d for 14 d (19). The sham and model groups administered saline solution for 14 d. This study was ethically approved by Changchun University of TCM.

### 2.5 Rat serum myocardial enzyme detection

The blood samples were centrifuged at 3000 rpm centrifugation for 10 min to get the serum. Creatine kinase (CK), aspartate transaminase (AST), myocardial troponin I (cTnI) levels were analyzed using the Mindray BS-420 Biochemical Analyzer (Rayto, Chemray).

### 2.6 TTC staining

The rats were anesthetized using 1% sodium pentobarbital at a dose of 45 mg/kg, followed by heart exercising, which was placed in a saline solution. The heart was frozen at -80 °C for 5 min and subsequently divided into five parts. The sections were stained with TTC and fixed in 4% paraformaldehyde.

### 2.7 HE staining and Masson staining

Myocardial tissue was fixed, embedded at -20 °C, and cut into 3 μm sections. Sections were stained with hematoxylin for 3–6 min, followed by 0.5% eosin for 2–3 min. After staining, samples were mounted using neutral gum. Imaging was performed with a microscope (ECLIPSE Ci, Nikon, Japan), and data acquisition was carried out using the Leica Application Suite system (Nikon DS-U3, Japan).

Paraffin sections of myocardial tissue were stained with Weigert hematoxylin, ponceau acid fuchsin and phosphomolybdic acid solution for differentiation. Aniline blue was used as a counterstain. The sections were dehydrated and made transparent with alcohol and xylene, following the same procedure as hematoxylin and eosin (HE) staining.

### 2.8 Bulk RNA-seq

Total RNA was extracted from myocardial tissue using the TRIzol splitting method (n = 3). mRNA was enriched using Oligo (dT), followed by fragmentation and cDNA synthesis. A library was constructed and subjected to quality control. The high-throughput sequencing was performed on a DNBSEQ-T7 platform (PE150) by Wuhan Lingsi Biotechnology Co., Ltd. The raw reads were used to identify differentially expressed genes (DEGs).

### 2.9 Cell culture

H9C2 cells (LJS-r012, Wuhan Lingjiesi) were cultured in DMEM. and passaged at ratios of 1:2:3 under standard conditions. Lipofectamine 2000 was used to transfect si-Hoxb4 siRNA and OV-Hoxb4 plasmid for 24 h. Thereafter, the cells were subjected to OGD/R for 2 h, followed by 24 h of culture with different concentrations of XBTYF. Additionally, the Wnt/β-catenin inhibitor IWR-1 (10 µM) (20) was added to the 96-well plates 30 min before 2 h OGD/R treatment, followed by culture with different concentrations of XBTYF for 24 h.

### 2.10 Cell counting kit-8 cell viability assay

1 × 10^4^ cells/well H9C2 cells were cultured in a 96-well plate, with six wells per group, overnight. To each well, 10 μL CCK-8 solution was added and incubated at 37 °C for 1 h. Subsequently, the absorbance was at 450 nm and the data were documented.

### 2.11 Western blot analysis

As described before, cell protein expression was analyzed via western blot (WB) (21). The primary antibodies were anti-Hoxb4 (ab133521, Abcam, 1:1000, Cambridge, MA, UK), anti-Bax (AF0120, Affinity, 1:1000, Jiangsu, China), anti-Bcl-2 (BF9103, Affinity, 1:1000, Jiangsu, China), cleaved-caspase 3 (Asp175), p17 (BF0711, Affinity, 1:1000, Jiangsu, China), anti-WNT4 (A7809, Abclonal, 1:1000, Wuhan, China), anti-WNT10B (A16717, Abclonal, 1:1000, Wuhan, China), β-catenin monoclonal (BF8016, Affinity, 1:2000, Jiangsu, China), c-Myc mouse monoclonal (BF8036, Affinity,1:1000, Jiangsu, China), and rabbit poly anti-GAPDH (AB-P-R 001, Goodhere, 1:1000, Hangzhou, China). After the membranes were washed three times and incubated overnight at 4 °C, the membranes were incubated with primary antibodies for 1 h at room temperature.

### 2.12 EdU and Calcein-AM/PI staining

1 × 10^5^ cells/well H9C2 cells were cultured in a 24-well plate. Each well received 10 μM EdU and was incubated for 2 h. After incubation, discard the EdU medium and fix the cells with paraformaldehyde. Permeate with 0.3% Triton X-100 and then incubate with Click-iT Additive Solution for 30 min. Nuclear staining was carried out using 300 μL of DAPI solution.

For Calcein-AM/PI staining, following PBS rinsing, cells were washed twice with 1× Assay Buffer. Calcein-AM was then added and incubated for 20 min at 37 °C in the dark, together with 3-5 μL of the original PI solution. After an additional 5 min incubation in darkness.

### 2.13 Molecular docking

The 3D structures of the bioactive compounds ginsenoside Rg3, protopine, papaverine, and ligustilide were obtained from the PubChem database of organic small molecules (https://pubchem.ncbi.nlm.nih.gov/). The 3D structure of the Hoxb4 protein was acquired from SWISS-MODEL (https://swissmodel.expasy.org/). Using PyMOL software, water molecules and inactive small molecule ligands were removed from the protein receptor structure. Molecular docking was subsequently performed using AutoDock to determine the binding affinity and binding positions between the active molecules of the drug and the receptor protein. The binding affinity (Affinity) is inversely related to the strength of the ligand-receptor interaction: the smaller the Affinity, the more stable the binding. An Affinity value of < -4.25 kcal/mol indicates a certain level of binding activity, while an Affinity of < -5.0 kcal/mol represents good binding activity, and an Affinity < -7.0 kcal/mol indicates strong binding activity.

### 2.14 Statistical analysis

All results were presented as mean ± SD. GraphPad Prism 8.0 software was used for data analysis. Unpaired t-tests were used to compare two groups, while one-way ANOVA was applied for comparisons among multiple groups. *p* < 0.05 was considered significant difference.

## 3 Results

### 3.1 Identification of active components of XBTYF

**Figure 1:**
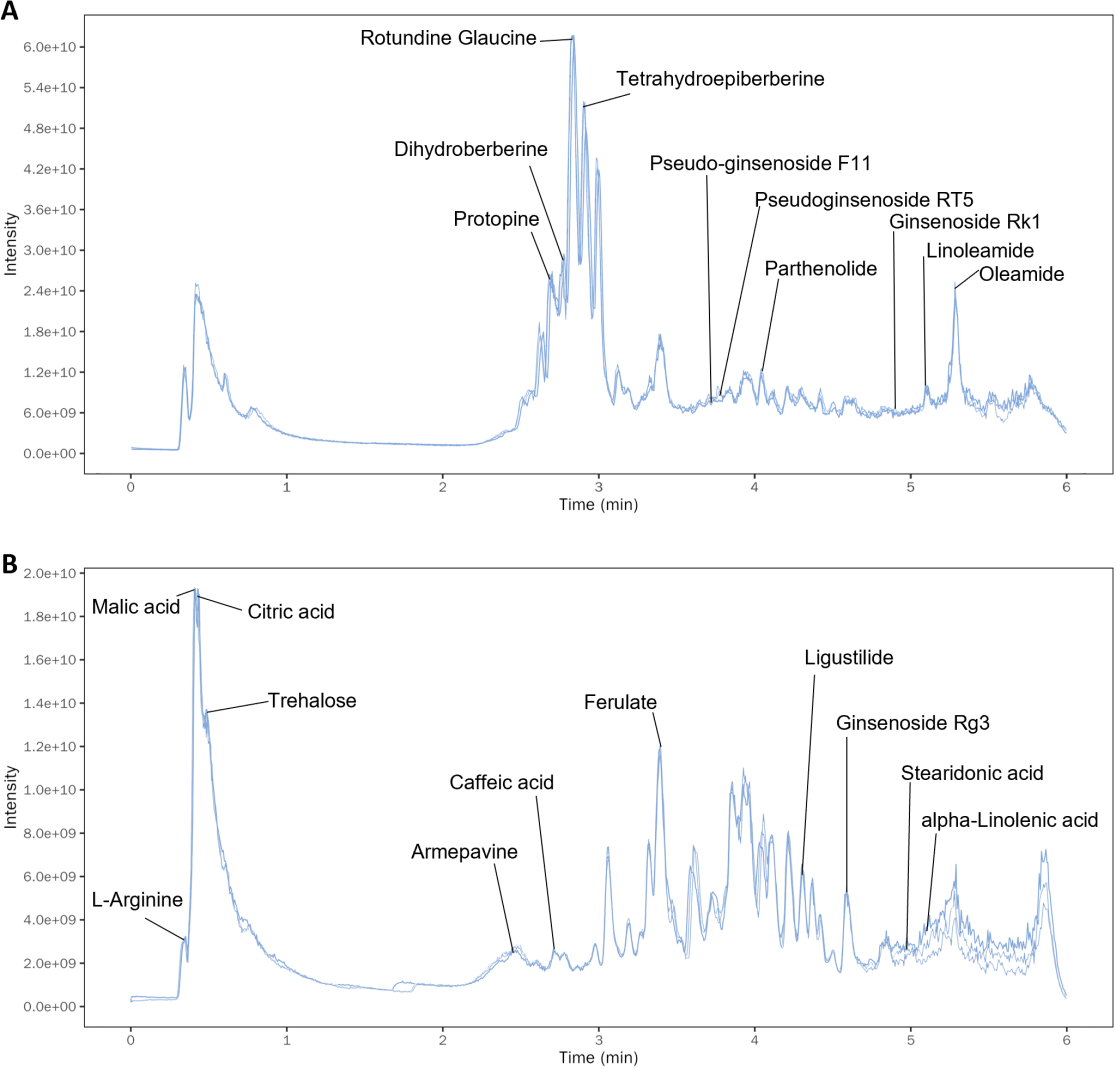
Identification of active components of XBTYF. **(A)** Total ion chromatograms (TIC) of XBTYF in positive ion mode. **(B)** TIC of XBTYF in negative ion mode.

A total of 614 compounds were identified based on molecular formula prediction and MS² fragmentation patterns, and the representative total ion chromatogram (TIC) is shown in **Figure 1**. The detected metabolites primarily included fatty acids and conjugates, small peptides, sesquiterpenoids, phenolic acids (C6-C1), and phenylpropanoids (C6-C3). Among them, ginsenoside Rg3 was identified as a characteristic secondary metabolite of *Ginseng Radix et Rhizoma* and *Notoginseng Radix*; protopine and papaverine were representative alkaloids of *Corydalis Rhizoma*; and *ligustilide* was a marker compound derived from *Chuanxiong Rhizoma* (**Supplementary Table 1**). The TIC also revealed additional metabolites with potential relevance to myocardial MIRI, including Ferulate, Stearidonic acid, Linoleamide, Armevine, and Trehalose, which have been previously linked to protective effects in cardiovascular conditions. These findings support the complex composition of the traditional formulas used for MIRI and suggest that the identified compounds may contribute to their therapeutic effects by modulating key metabolic pathways associated with ischemic injury, oxidative stress, and inflammation.

### 3.2 XBTYF protects MIRI rats from myocardial injury

**Figure 2:**
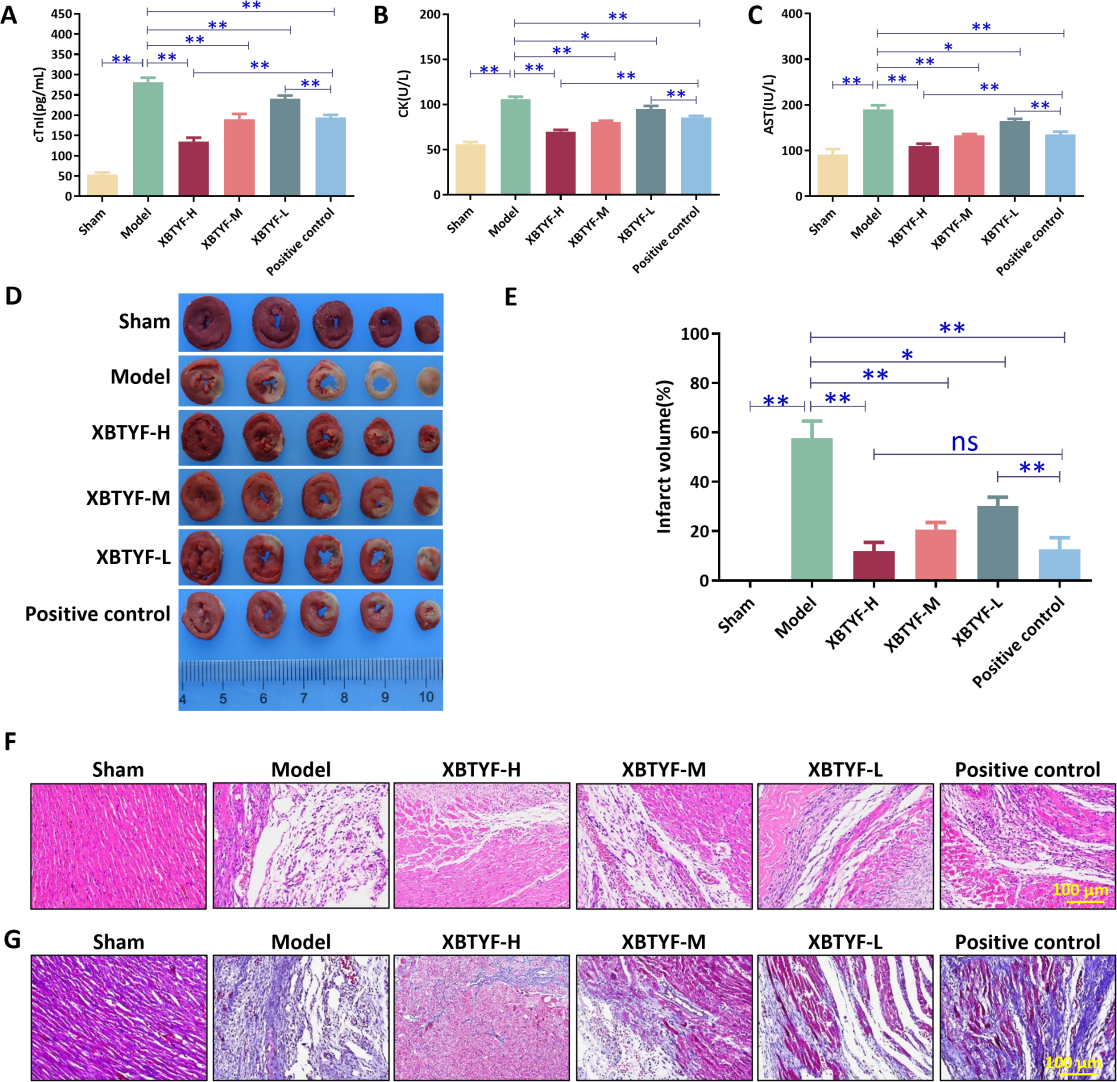
The protective effect of XBTYF in MIRI rats from myocardial injury. **(A-C)** Biochemical analysis of serum AST, CK, cTnl content in rats. **(D, E)** Pathological tissue of myocardium using TTC staining and area analysis of myocardial infarction. **(F)** HE staining of myocardial tissue. **(G)** MASSON staining of myocardial tissue. *p < 0.05, **p < 0.01, n = 3. Scale bar: 100 μm. Positive control: Qishen Yiqi Droplet.

CK, AST, and cTnI were recognized as key biomarkers of myocardial injury. In the model group, the serum levels of CK, AST, and cTnI were significantly higher than those in the sham group (*p* < 0.01). Treatment with XBTYF led to a concentration-dependent manner, similar to the positive control group. The low dose of XBTYF treatment showed increased levels of CK, AST, and cTnI compared to the positive control group. In contrast, the XBTYF-H (high concentration) group exhibited significantly lower levels (*p* < 0.01) compared to the positive control group, indicating that XBTYF can ameliorate MIRI (**Figures 2A–C**). TTC staining showed a noticeable increase in the myocardial infarction area in the model group relative to the sham group. XBTYF reduced the myocardial infarction area in a concentration-dependent manner, with effects similar to the positive control group. Compared to the positive control group, the myocardial infarction areas in the XBTYF-H and XBTYF-M groups showed no significant differences, while the XBTYF-L group exhibited an increased infarct size, indicating that higher concentrations of XBTYF can effectively suppress myocardial infarction (**Figures 2D, E**). HE staining results indicated normal myocardial tissue in the sham group, disorganized structure, and cell necrosis in the model group, whereas the XBTYF and positive control groups showed improved fiber structure, particularly in the XBTYF-H group, which showed the most significant improvement (*p* < 0.01). Masson staining results demonstrated regular cell distribution in the sham group and necrosis in the model group, whereas XBTYF and the positive control group ameliorated myocardial fibrosis, especially in the XBTYF-H group (**Figures 2F, G**). These findings suggested that XBTYF can protect MIRI rats from myocardial injury.

### 3.3 XBTYF influences signal pathway and gene expression

**Figure 3:**
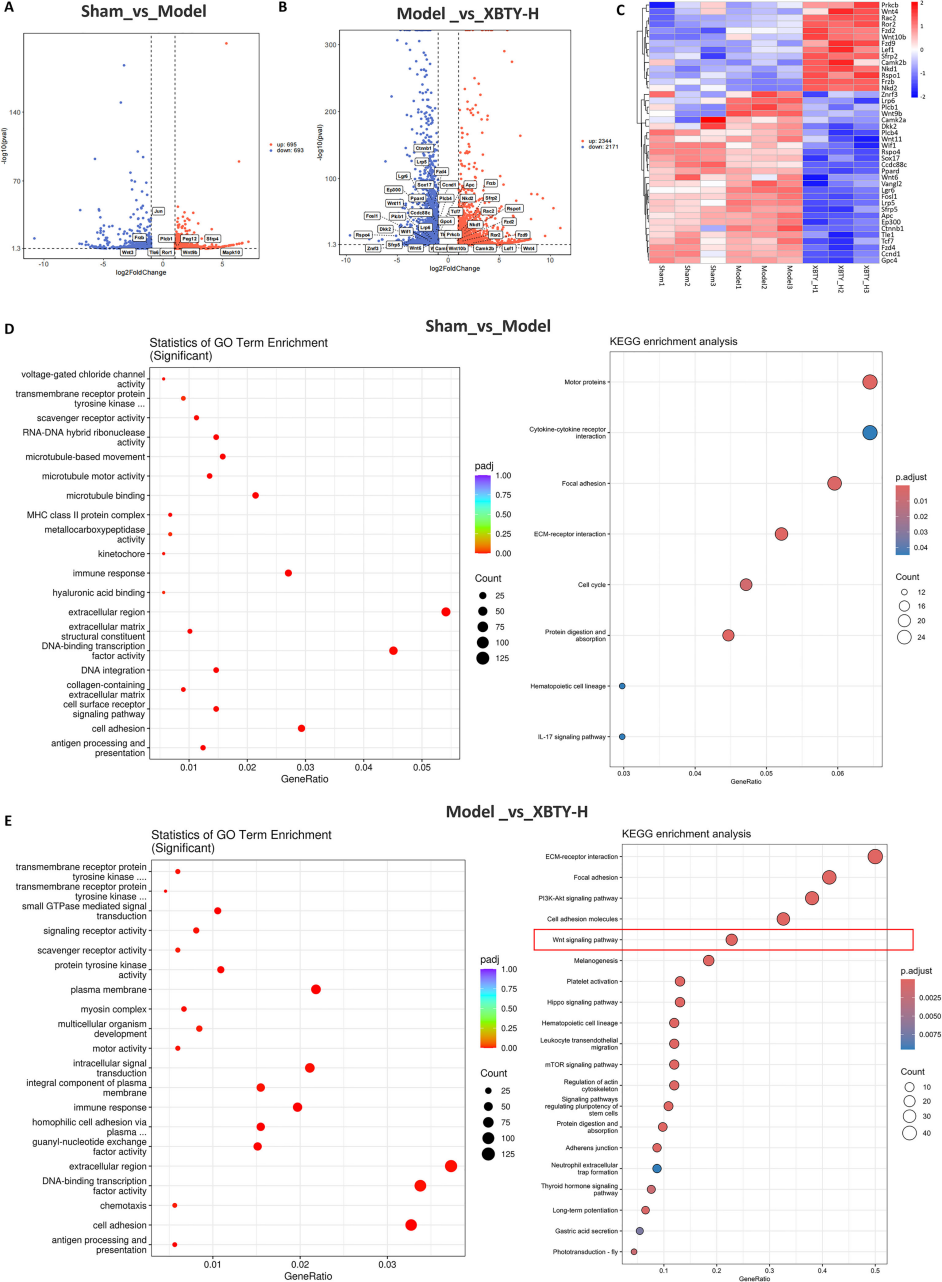
Expression profiles of Wnt signaling genes revealed by RNA-seq. **(A, B)** Volcano plot of DEGs among Sham, model, and XBTYF-H group; **(C)** Heat-map of DEGs associated with the Wnt signaling pathway; **(D, E)** GO and KEGG enrichment analysis of differential gene expression. n = 3.

To systematically investigate the mechanism of XBTYF, GO and KEGG pathway enrichment analyses were performed. We compared gene expression differences between the Sham and Model groups, as well as between the Model and XBTY-H groups. In the Sham versus Model comparison, 1,388 DEGs were detected, with 695 transcripts up-regulated and 693 down-regulated. In contrast, the Model versus XBTYF-H comparison yielded 4,515 DEGs, including 2,344 up-regulated and 2,171 down-regulated (**Figures 3A, B**). Strikingly, among these XBTYF-responsive DEGs, we observed a coordinated change in the expression of multiple core components of the Wnt signaling pathway. This is visually summarized in the heatmap (**Figure 3C**, **Supplementary Figure 1**), which shows that XBTYF-H treatment consistently altered the expression of key Wnt-related genes, including pronounced transcriptional alterations of Wnt4 and Wnt10b relative to the Model group.

In the comparison between the Sham and Model groups, GO analysis revealed significant enrichment in biological processes such as voltage-gated chloride channel activity, immune response, and antigen processing and presentation in the Model group. KEGG analysis further highlighted enriched pathways, including cytokine-cytokine receptor interaction, focal adhesion, and IL-17 signaling pathway (**Figure 3D**). In the comparison between the Model and XBTY-H groups, GO analysis identified significant enrichment in processes like immune response, multicellular organism development, and extracellular matrix organization. KEGG analysis revealed significant enrichment in pathways such as Wnt signaling pathway, ECM receptor interaction, and PI3K-Akt signaling pathway, suggesting that XBTY-H may regulate these pathways to influence cell function and immune responses (**Figure 3E**).

**Figure 4:**
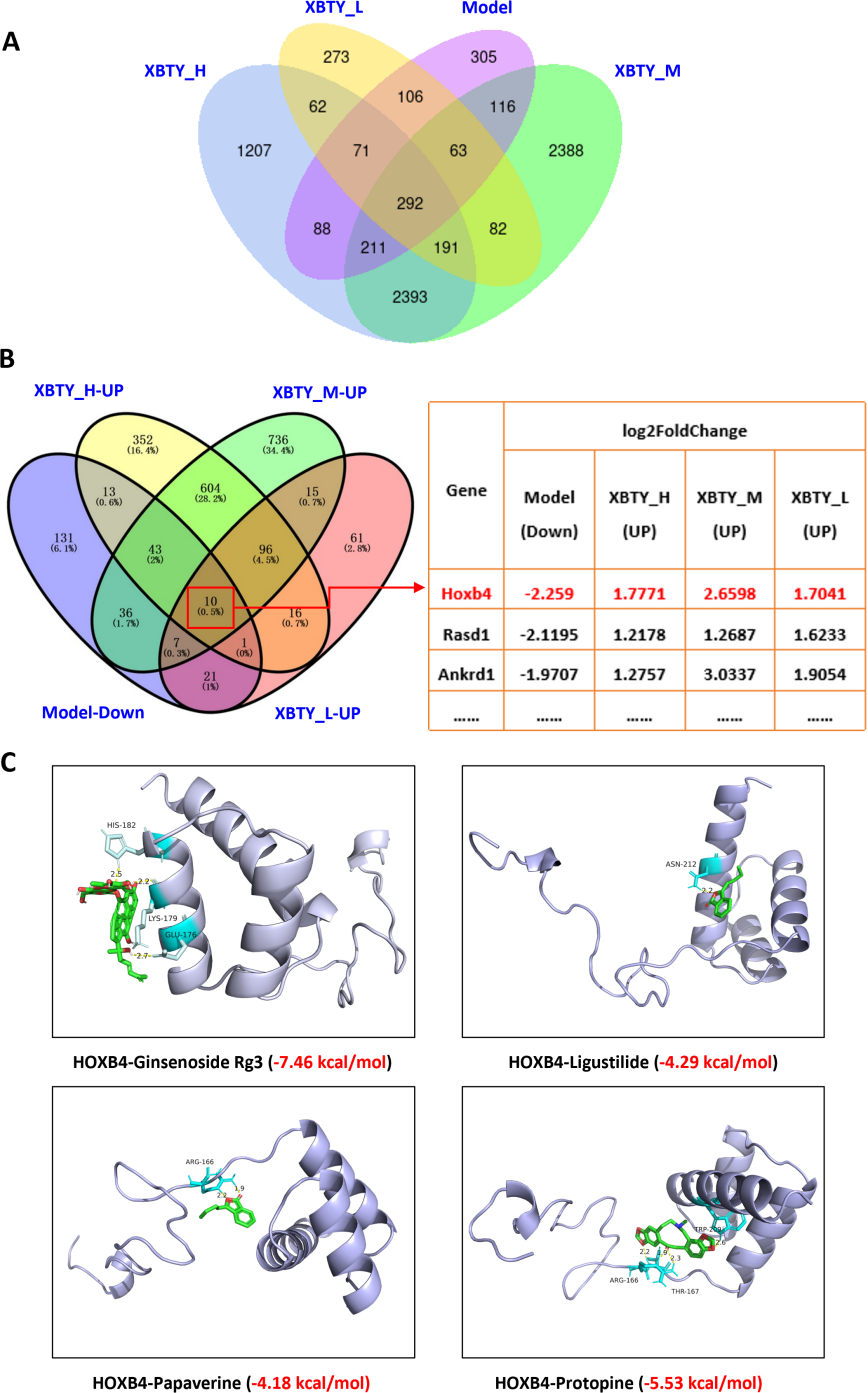
Summary of specific gene analysis and molecular docking analysis. **(A)** Venn diagram for DEGs analysis; **(B)** Venn diagram for specific gene analysis in XBTYF; **(C)** Molecular docking results of HOXB4 and XBTY active components.

Further analysis identified 10 specific genes associated with XBTYF: *Sphk1*, *Cpxm2*, *Nppb*, *Nppa*, *Ankrd23*, *Myh7*, *Ankrd1*, *Rasd*1, *Hoxb4*, and *Serpina3n*. Among these, Hoxb4 was significantly decreased in the model group, whereas its expression increased in each XBTYF group (**Figures 4A, B**). Molecular docking were performed to investigate the binding affinities and interactions of ginsenoside Rg3, protopine, papaverine, and ligustilide with the Hoxb4 protein (**Figure 4C**). The results indicate varying degrees of binding affinity for each compound. Ginsenoside Rg3 exhibited the strongest binding affinity of -7.46 kcal/mol, suggesting a moderate but significant interaction with Hoxb4. The key residues involved in this interaction include HIS-182, GLU-176, and LYS-179, which play crucial roles in stabilizing the ligand-protein complex. In contrast, ligustilide demonstrated a lower binding affinity of -4.29 kcal/mol, reflecting a relatively weaker binding interaction with Hoxb4. The interaction was primarily mediated by ASN-212, which is critical for the binding stability. Similarly, papaverine also showed a moderate binding affinity of -4.18 kcal/mol, with ARG-166 being the key residue involved in the ligand-protein interaction. Protopine, with a binding affinity of -5.53 kcal/mol, displayed a moderate binding capacity to Hoxb4. The residues ARG-166 and THR-167 were found to interact with protopine, further suggesting potential for modulating Hoxb4 activity.

Overall, the molecular docking results highlight that ginsenoside Rg3 has the strongest binding affinity for Hoxb4, followed by protopine, ligustilide, and papaverine. These findings provide insight into the potential of these compounds to interact with Hoxb4, with ginsenoside Rg3 exhibiting the most robust binding potential, indicating its significant role in modulating Hoxb4 activity.

### 3.4 XBTYF enhances Hoxb4 to inhibit oxygen and glucose-deprivation-induced H9C2 cell apoptosis with relationship to Wnt/β-catenin pathway

**Figure 5:**
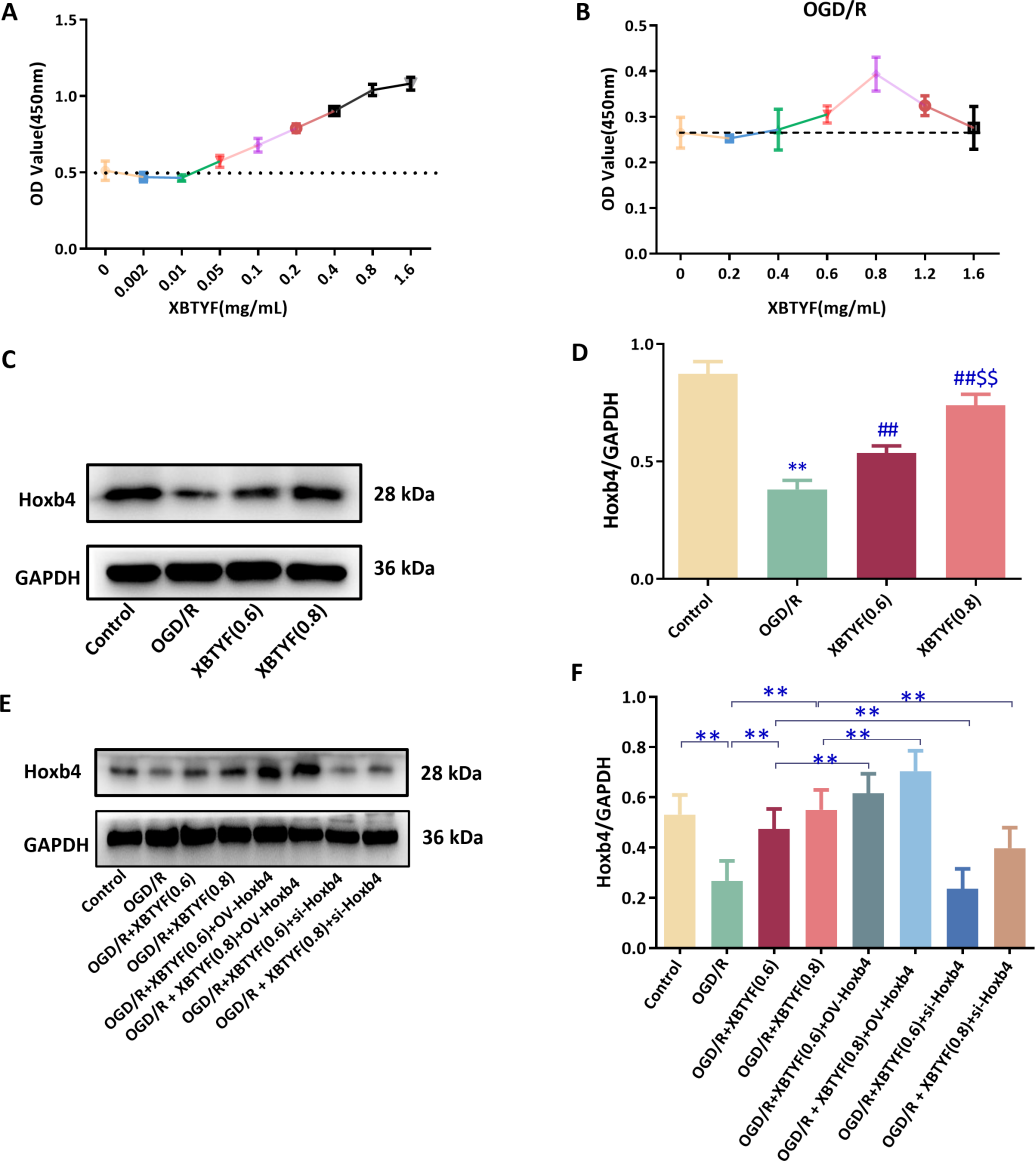
The effect of XBTYF in H9C2 cell viability and its relationship with Hoxb4. **(A)** CCK-8 assay results for different concentrations of XBTYF in normal H9C2 cells. **(B)** CCK-8 assay results for different concentration of XBTYF in OGD/R H9C2 cells. **(C, D)** WB analysis of Hoxb4 expression influenced by OGD/R and XBTYF treatment. Compared with control group, **p < 0.01; compared with OGD/R group, ##*p* < 0.01; compared with XBTYF group (0.6mg/mL), $$*p* < 0.01, n = 3. **(E, F)** WB analysis evaluating the effect of XBTYF on Hoxb4 expression under the OGD/R condition in H9C2 cells, **p < 0.01, n = 3. 0.6: 0.6 mg/mL, 0.8: 0.8 mg/mL.

CCK-8 assay indicated that XBTYF significantly promoted cell viability at concentrations of 0.5-1.6 mg/mL (**Figure 5A**). In OGD/R cells, 0.6 and 0.8 mg/mL of XBTYF significantly inhibited cell damage and promoted proliferation (**Figure 5B**). WB showed that Hoxb4 expression was significantly lower in OGD/R cells, whereas 0.6 mg/mL and 0.8 mg/mL XBTYF significantly enhanced Hoxb4 expression (**Figures 5C, D**). In OGD/R and OV-Hoxb4 conditions, 0.8 mg/mL XBTYF exhibited higher Hoxb4 expression than 0.6 mg/mL. The si-Hoxb4 expression resulting from si-Hoxb4 transfection was reversed by XBTYF administration (**Figures 5E, F**; **Supplementary Figure 2**).

**Figure 6:**
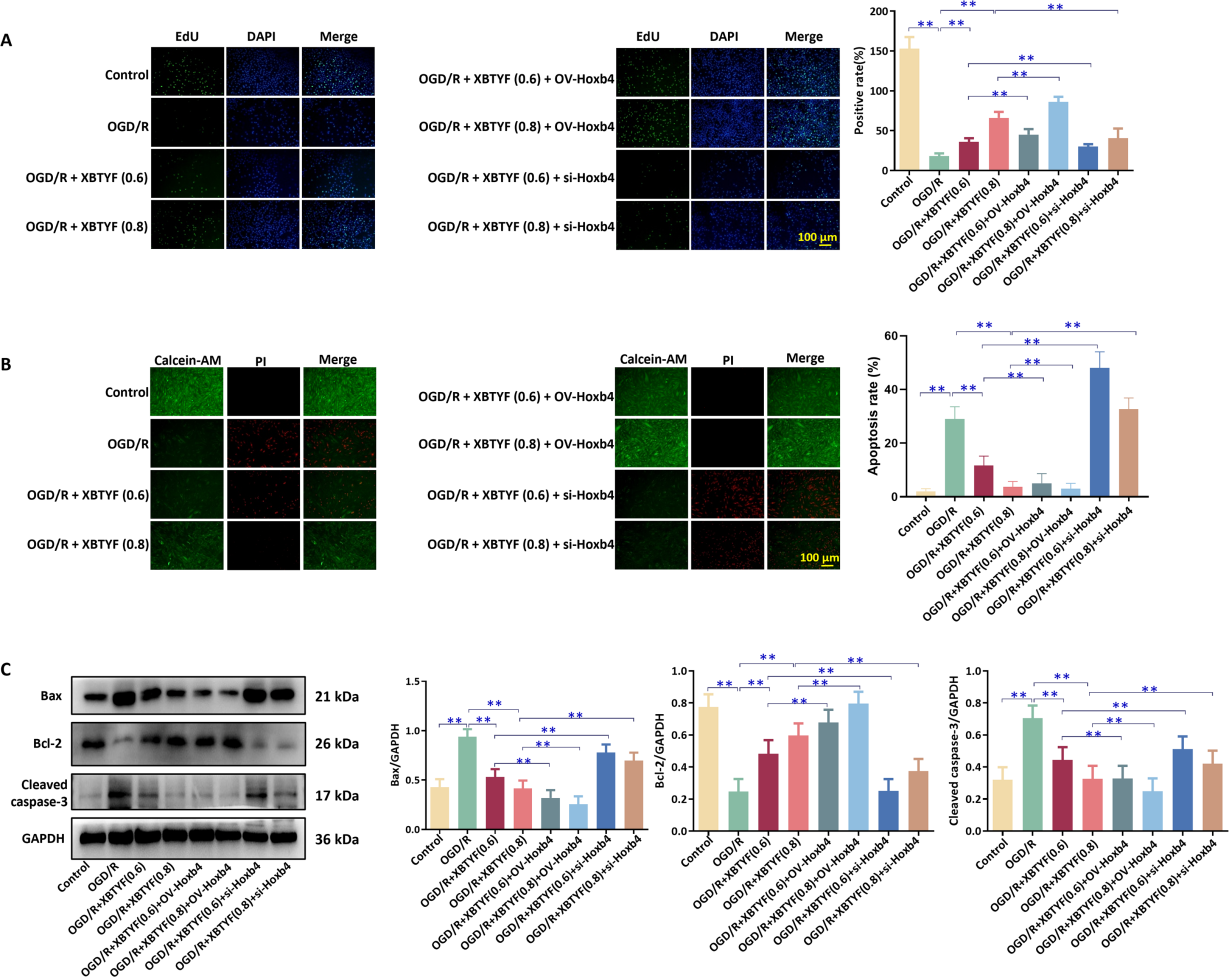
The effect of XBTYF on Hoxb4 and its influence on OGD/R induced cell apoptosis. **(A)** EdU test results showing H9C2 cell proliferation; **(B)** Calcein-AM/PI staining results indicating cell apoptosis; **(C)** WB analysis illustrating the expression of Bax, cleaved caspase-3, and Bcl-2 proteins. *p < 0.05, **p < 0.01, n = 3. Scale bar: 100 μm, 0.6: 0.6 mg/mL, 0.8: 0.8 mg/mL.

EdU results showed that cell proliferation in the OGD/R group was significantly reduced compared to that in the control group. Under OGD/R conditions, 0.6 and 0.8 mg/mL of XBTYF promoted cell proliferation, and OV-Hoxb4 enhanced this effect. In si-Hoxb4, the effect of XBTYF was inhibited (**Figure 6A**). Calcein-AM/PI staining revealed that apoptosis significantly increased in the OGD/R group compared to that in the control group. Under OGD/R conditions, 0.6 and 0.8 mg/mL XBTYF reduced cell apoptosis, and OV-Hoxb4 enhanced this inhibition. In si-Hoxb4, the protective effect of XBTYF was reduced (**Figure 6B**).

WB indicated that the OGD/R group had significantly enhanced expression of Bax and cleaved caspase-3, whereas Bcl-2 expression was significantly reduced (*p* < 0.01). XBTYF intervention reversed the effect of OGD/R. Furthermore, OV-Hoxb4 enhanced the inhibition of Bax and cleaved caspase-3 and the promotion of Bcl-2 by 0.6 and 0.8 mg/mL XBTYF. In contrast, si-Hoxb4 reduced the effect of XBTYF. Overall, these results suggest that XBTYF upregulates Hoxb4 expression, inhibits the expression of Bax and cleaved caspase-3, and enhances Bcl-2 expression, thereby promoting cell proliferation, suppressing apoptosis, and ultimately protecting cardiac myocytes (**Figure 6C**).

**Figure 7:**
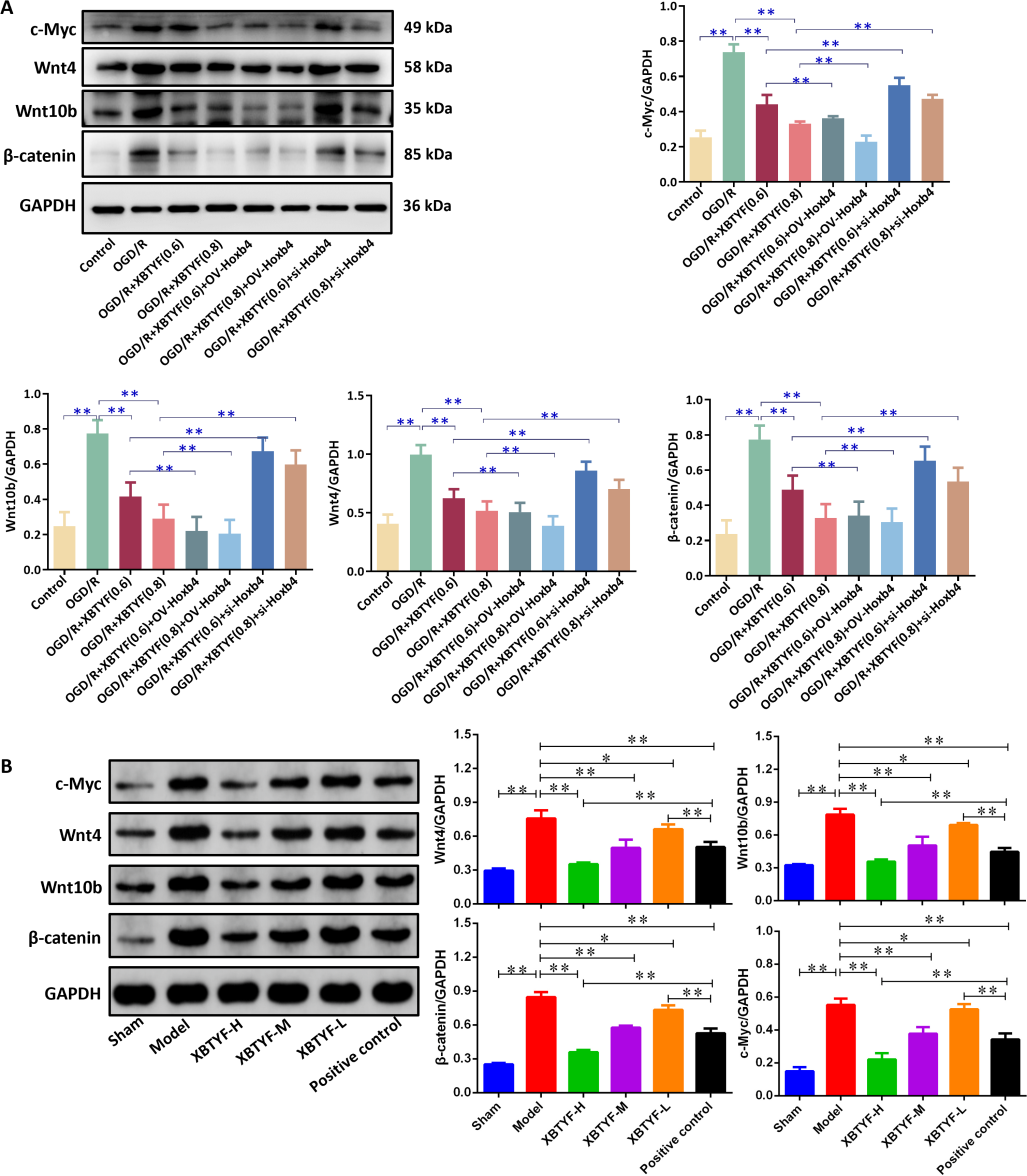
WB of Wnt/β-catenin pathway proteins regulated by XBTYF via Hoxb4. **(A)** H9C2 cells; **(B)** The myocardial tissue of rats. **p < 0.01, n = 3, 0.6: 0.6 mg/mL, 0.8: 0.8 mg/mL.

Meanwhile, WB results indicated that the OGD/R group had significantly higher expression of Wnt signaling pathway-related proteins c-Myc, Wnt10b, Wnt4, and β-catenin than the control group in H9C2 cells (*p* < 0.05). Under OGD/R conditions, the XBTYF group (0.6 and 0.8 mg/mL) significantly reduced the levels of these proteins (*p* < 0.05), with 0.8 mg/mL showing better effects than 0.6 mg/mL. OV-Hoxb4 exhibited lower expression, whereas si-Hoxb4 reversed this effect (**Figure 7A**). *In vivo* experiments showed that, compared with the sham group, the Model group exhibited significantly increased expression levels of Wnt4, Wnt10b, β-catenin, and c-Myc (*p* < 0.01). Compared with the Model group, treatment with XBTYF significantly reduced the expression of Wnt4, Wnt10b, β-catenin, and c-Myc in the XBTYF-L, XBTYF-M, and XBTYF-H groups, showing a dose-dependent effect. Similarly, compared with the Model group, the positive control group showed markedly decreased expression levels of Wnt4, Wnt10b, β-catenin, and c-Myc (*p* < 0.01). Compared with the positive control group, the XBTYF-L group exhibited significantly higher expression levels of these proteins (*p* < 0.01), whereas the XBTYF-H group showed significantly lower expression levels (*p* < 0.01) (**Figure 7B**). These results indicate that XBTYF downregulates Wnt4, Wnt10b, β-catenin, and c-Myc expression in a dose-dependent manner.

### 3.5 XBTYF enhances Hoxb4 to inhibit Wnt/β-catenin signal pathway in OGD/R leading H9C2 cell apoptosis

**Figure 8:**
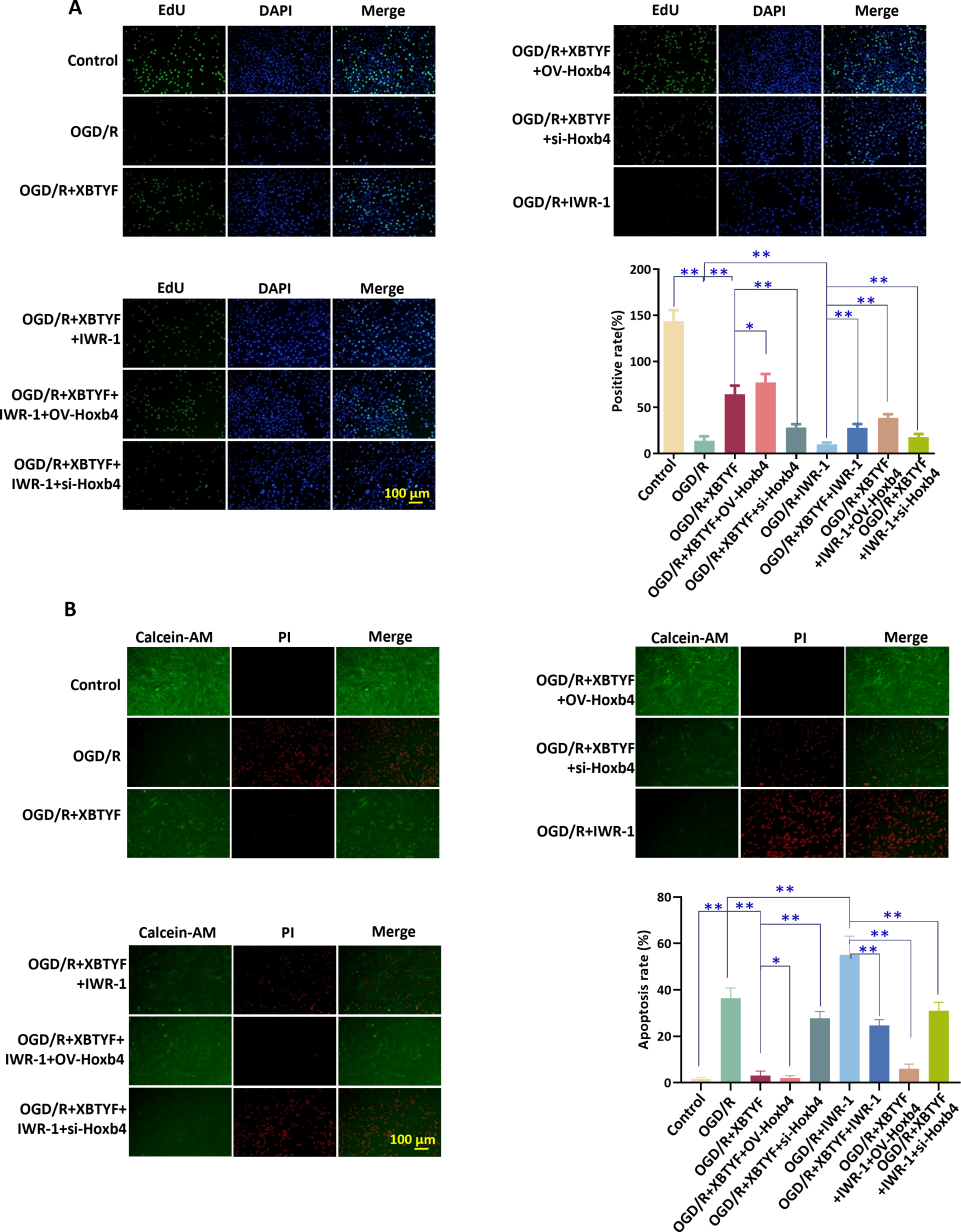
XBTYF regulates Hoxb4-dependent activation of the Wnt/β-catenin pathway and attenuates apoptosis in OGD/R-injured H9C2 cells. **(A)** EdU assay for assessing H9C2 cell proliferation, **(B)** Calcein-AM/PI for evaluating cell apoptosis. XBTYF: 0.8 mg/mL, IWR-1: 10 μM. *p < 0.05, **p < 0.01, n = 3. Scale bar: 100 μm.

EdU results indicated that cell proliferation was inhibited in the OGD/R group. Under OGD/R conditions, adding the Wnt/β-catenin inhibitor IWR-1 promoted cell proliferation, and combining it with XBTYF enhanced this effect. OV-Hoxb4 led to further increases in proliferation, whereas si-Hoxb4 significantly reduced it (**Figure 8A**). Calcein-AM/PI staining showed that cell apoptosis was promoted in the OGD/R group. Under OGD/R conditions, adding IWR-1 inhibited cell apoptosis, and combining it with XBTYF enhanced this inhibitory effect. OV-Hoxb4 resulted in further inhibition, whereas si-Hoxb4 significantly accelerated apoptosis (**Figure 8B**).

**Figure 9:**
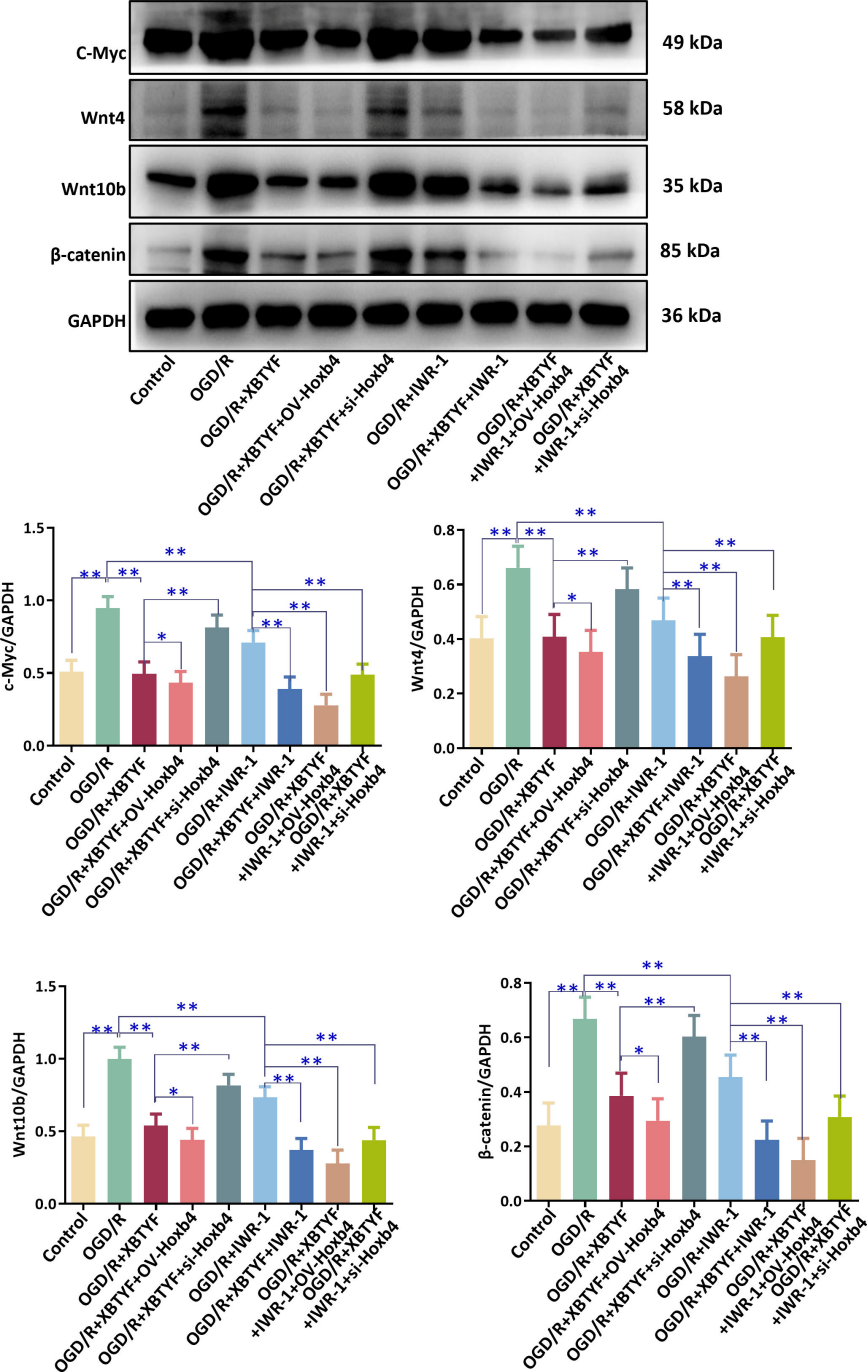
WB analysis of Wnt/β-catenin pathway in H9C2 cells after OGD/R injury, showing the effect of XBTYF via Hoxb4 on Wnt/β-catenin pathway activation. XBTYF: 0.8 mg/mL, IWR-1: 10 μM. *p < 0.05, **p < 0.01, n = 3.

In the OGD/R group, the expression levels of Wnt/β-catenin pathway-associated proteins, including c-Myc, Wnt10b, Wnt4, and β-catenin, were significantly higher than those in the control group (*p* < 0.01). Under OGD/R conditions, adding IWR-1 reduced protein expression, and combining it with XBTYF further lowered the expression. OV-Hoxb4 showed further reduction, whereas si-Hoxb4 significantly increased expression (*p* < 0.01) (**Figure 9**).

Overall, XBTYF enhances Hoxb4 expression, promotes cell proliferation, and inhibits cell apoptosis, playing a key role in the Wnt/β-catenin signaling pathway.

## 4 Discussion

MIRI commonly occurs during clinical procedures such as percutaneous coronary intervention, intravenous thrombolysis, heart transplantation, and coronary artery bypass grafting. It triggers a cascade of pathological events, including endothelial dysfunction, vascular smooth muscle injury, microvascular impairment, and tissue damage. These alterations may lead to transient increases in vascular resistance, persistent capillary obstruction, and even the no-reflow phenomenon, thereby compromising the restoration of coronary blood flow (22). Current strategies for MIRI management include both pharmacological and non-pharmacological approaches. Although these interventions provide some benefits, their efficacy remains limited (23, 24). In contrast, TCM, characterized by its multi-component and multi-target properties, offers a more integrated regulatory effect on complex pathological processes (25). Therefore, exploring specific TCM-based interventions may help address existing therapeutic gaps and provide safer and more effective treatment options.

XBTYF alleviates mitochondrial dysfunction and promotes angiogenesis in cardiomyocytes, thereby preserving cardiac function under ischemic conditions (18). In the present study, XBTYF significantly reduced infarct size, inhibited inflammatory cell infiltration and fibrosis, and decreased serum levels of CK, AST, and cTnI. RNA-seq revealed that XBTYF promotes Hoxb4 expression. Inhibiting Hoxb4 expression exacerbates cell damage under OGD/R conditions, and this damage cannot be fully reversed by XBTYF. The Homeobox (HOX) gene family comprises highly conserved transcription factors that play essential roles in development. Among them, Hoxb4 regulates hematopoietic stem cell activation, differentiation, proliferation, and morphology (26). Hoxb4 may also serve as a biomarker for acute myocardial infarction (27), and its overexpression can inhibits myocardial cell apoptosis. In the present study, CCK-8 assay demonstrated that XBTYF attenuated OGD/R-induced cellular injury. Moreover, Hoxb4 overexpression enhanced the protective effect of XBTYF, whereas Hoxb4 knockdown diminished it. These findings collectively suggest that XBTYF promotes Hoxb4 expression, thereby inhibiting OGD/R-induced apoptosis, stimulating H9C2 cell proliferation, and reducing cellular injury.

Comprehensive metabolite profiling revealed 614 chemical entities in XBTYF, including fatty-acid conjugates, small peptides, sesquiterpenoids, C6-C1 phenolic acids and C6-C3 phenylpropanoids. This chemical diversity provides a plausible substrate for the multi-target protective effects of XBTYF against MIRI. Five dammarane-type saponins—ginsenosides Rg3, RT5, Rk1, F11 and Rg6—were recovered, chemically confirming the presence of Ren Shen and San Qi. Rg3 in the formulation. Ginsenoside Rg3 has been reported to attenuate post-ischemic apoptosis and inflammation, thereby preserving cardiac function (28). Although direct MIRI data for RT5, Rk1, F11 and Rg6 remain limited, structurally related ginsenosides consistently ameliorate oxidative stress and calcium overload in ischemic myocardium, suggesting that these additional saponins may act in concert with Rg3 to reinforce antioxidant and anti-apoptotic defenses.

In addition, alkaloids such as protopine and papaverine were identified. Protopine has been shown to protect H9C2 cardiomyocytes from hypoxia/reoxygenation injury by reducing oxidative stress and activating the PTEN/PI3K/Akt pathway (29), while papaverine improves microvascular perfusion and attenuates the no-reflow phenomenon following myocardial infarction (30). Ligustilide, the diagnostic phthalide of Chuan Xiong, was also detected. Recent study demonstrates that ligustilide covalently activates muscular creatine kinase and thereby raises myocardial phosphocreatine (31) and limiting infarct expansion in acute ischemia. This energy-preserving mechanism adds a metabolic dimension to XBTYF’s cardioprotection. Collectively, these signature metabolites intervene at complementary nodes of MIRI pathology—oxidative injury (ginsenosides), microvascular obstruction (papaverine), intracellular signaling and autophagy (protopine), and high-energy phosphate buffering (ligustilide). Their simultaneous presence in XBTYF provides a solid chemical rationale for the transcriptomic finding that high-dose XBTYF re-engages canonical Wnt/β-catenin signaling, a pathway closely linked to cardiomyocyte survival and post-ischemic remodeling (32). Thus, the phytochemical composition of XBTYF aligns with and likely underpins the multi-level protection observed in both *in vivo* and *in vitro* models of MIRI.

KEGG analysis revealed that the Wnt/β-catenin signal pathway as one of the most significant affected pathway following XBTYF treatment. Wnt signaling is a growth stimulation factor promoting cell proliferation and directing tissue growth. Wnt signaling is generally categorized into the canonical (β-catenin-dependent) and non-canonical (β-catenin-independent) pathways (33). Activation of the Wnt/β-catenin pathway is closely related to myocardial remodeling, infarction, and arrhythmia (34, 35). Our results show that XBTYF significantly inhibits Wnt/β-catenin activation, reducing H9C2 cell damage under OGD/R conditions. Taken together, the RNA-seq data indicate that high-dose XBTYF induces broad transcriptional changes in Wnt-related genes in the post-ischemic myocardium. At the functional level, WB analysis confirmed that XBTYF treatment restored Wnt/β-catenin target protein levels toward Sham levels.

Hoxb4 expression changes can function via Wnt/β-catenin signaling pathway. On one hand, Hoxb4 can inhibit cervical cancer cell proliferation and tumorigenesis by downregulating Wnt/β-catenin activity (26). On the other hand, Hoxb4 overexpression has been shown to activate the Wnt/β-catenin pathway, promoting proliferation, migration, and angiogenesis in lipopolysaccharide (LPS)-stimulated cardiomyocytes, while alleviating LPS-induced apoptosis and vascular hyperpermeability (36). To clarify the underlying mechanism, we used the Wnt signal pathway inhibitor IWR-1, and OV-Hoxb4, si-Hoxb4 vector for coalition analysis. The results indicated that Hoxb4 overexpression significantly enhances the protective effect of XBTYF under OGD/R conditions. The Wnt/β-catenin inhibitor further strengthened the effect of XBTYF, whereas si-Hoxb4 markedly diminished it. These results suggest that XBTYF may exert its effects by upregulating Hoxb4 and modulating the Wnt/β-catenin pathway.

This study has several limitations. First, the sample size was relatively small, and larger studies are needed to validate these findings in future studies. Second, Calcein-AM/PI staining was used to assess cell viability, which does not precisely distinguish apoptotic stages; more specific methods such as Annexin V/PI flow cytometry should be applied in future studies. In addition, functional cardiac assessments were not included. The cardioprotective effects of XBTYF were mainly evaluated by serum myocardial enzymes and histopathological analyses (TTC, HE, and Masson staining), while key functional indicators, including echocardiography, ANP/BNP levels, heart weight indices, and survival analysis, were not assessed. Future work will incorporate additional cell death modalities and functional evaluations, including echocardiography, heart weight indices, and survival analysis, to further clarify the cardioprotective mechanisms of XBTYF.

Myocardial cell death plays a crucial role in MIRI, with previous studies focusing on apoptosis and necrosis mechanisms. New forms of cell death, such as ferroptosis, necroptosis, pyroptosis, have also been implicated in MIRI. These processes contribute to oxidative stress, calcium overload, and inflammatory responses, ultimately exacerbating myocardial injury and impairing cardiac function (37). This study explored the effects of XBTYF on myocardial cell apoptosis in MIRI. Further research will focus on other types of cell death to illustrate the comprehensive protective mechanism of XBTYF in MIRI.

## 5 Conclusion

Based on RNA-sequencing and *in vitro* experiments, a Wistar rat model was established to demonstrate that XBTYF alleviates MIRI and protects myocardial cells *in vivo*. Therefore, it was concluded that XBTYF upregulates Hoxb4 to inhibit Wnt/β-catenin signaling pathway protein expression, providing cardiomyocyte protection.

## Data Availability

We confirm that the LC-MS analysis of TCM and RNA-seq raw data have been deposited in the SCcienceDB repository. This data can be found here: 10.57760/sciencedb.34816; 10.57760/sciencedb.34834.

## Supplementary material

Supplementary Figure 1 Heatmap of all DEGs. **(A)** Heatmap of DEGs comparing the sham group with the model group, **(B)** Heatmap of DEGs comparing the high-dose XBTYF group with the model group, **(C)** Heatmap of DEGs comparing the low-dose XBTYF group with the model group, **(D)** Heatmap of DEGs comparing the medium-dose XBTYF group with the model group. J: Sham group, M: Model group, H: High-dose XBTYF group, MI: Medium-dose XBTYF group, L: Low-dose XBTYF group. n = 3.

Supplementary Figure 2 RT-qPCR analysis of si-Hoxb4 and OV-Hoxb4 expression in H9C2 cells. **p* < 0.05, ***p* < 0.01, n = 3.
