# Supplementary figures and images for: Hoxb4 upregulation by Xuan Bi Tong Yu Fang confers cardioprotection via repression of the Wnt/β-catenin pathway in myocardial ischemia-reperfusion injury
Source: Front Immunol. 2026 May 7;17:1767595. doi: 10.3389/fimmu.2026.1767595 (PMC13189895; doi:10.3389/fimmu.2026.1767595)

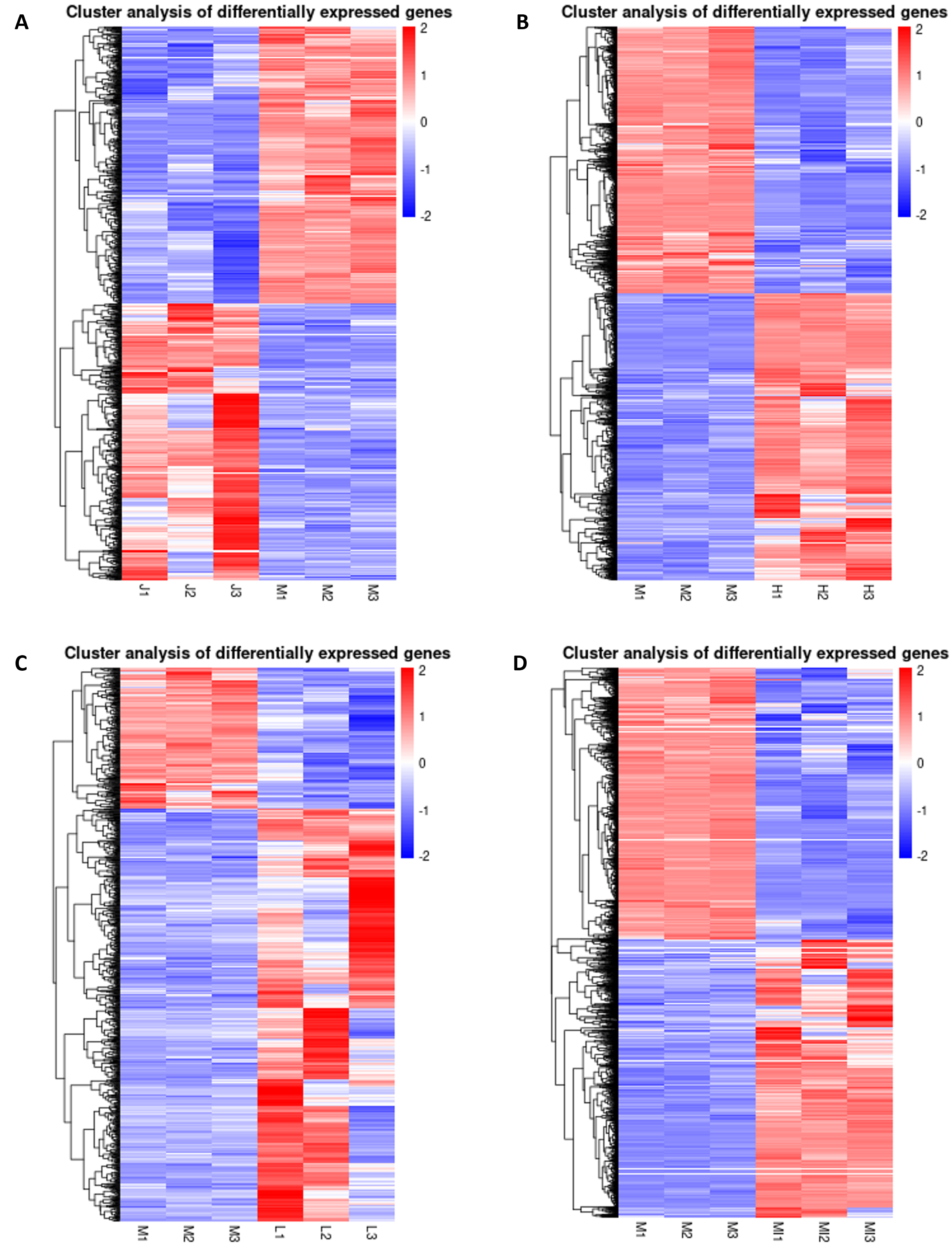

Supplement: Supplementary Figure 1 — Heatmap of all DEGs. (A) Heatmap of DEGs comparing the sham group with the model group, (B) Heatmap of DEGs comparing the high-dose XBTYF group with the model group, (C) Heatmap of DEGs comparing the low-dose XBTYF group with the model group, (D) Heatmap of DEGs comparing the medium-dose XBTYF group with the model group. J: Sham group, M: Model group, H: High-dose XBTYF group, MI: Medium-dose XBTYF group, L: Low-dose XBTYF group. n = 3. [file Image1.tif]

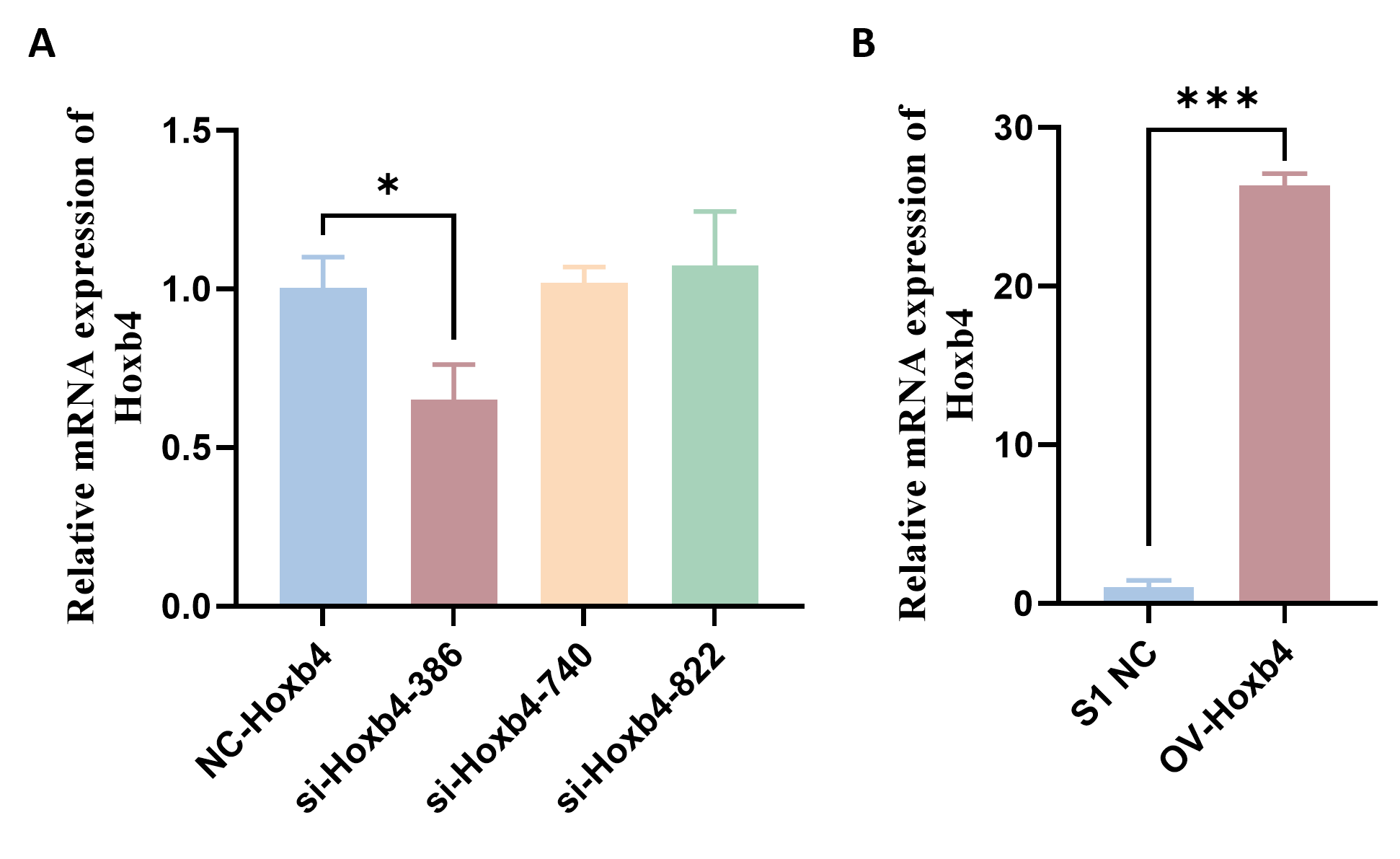

Supplement: Supplementary Figure 2 — RT-qPCR analysis of si-Hoxb4 and OV-Hoxb4 expression in H9C2 cells. *p < 0.05, **p < 0.01, n = 3. [file Image2.tif]
